# Avatars with faces of real people: A construction method for scientific experiments in virtual reality

**DOI:** 10.3758/s13428-021-01676-5

**Published:** 2021-09-09

**Authors:** Matthew C. Fysh, Iliyana V. Trifonova, John Allen, Cade McCall, A. Mike Burton, Markus Bindemann

**Affiliations:** 1 School of Psychology, University of Kent, Canterbury, CT2 7NP UK; 2 Department of Psychology, University of York, York, UK

**Keywords:** Face, Avatar, Virtual reality, Recognition, Matching, Face-space

## Abstract

Experimental psychology research typically employs methods that greatly simplify the real-world conditions within which cognition occurs. This approach has been successful for isolating cognitive processes, but cannot adequately capture how perception operates in complex environments. In turn, real-world environments rarely afford the access and control required for rigorous scientific experimentation. In recent years, technology has advanced to provide a solution to these problems, through the development of affordable high-capability virtual reality (VR) equipment. The application of VR is now increasing rapidly in psychology, but the realism of its avatars, and the extent to which they visually represent real people, is captured poorly in current VR experiments. Here, we demonstrate a user-friendly method for creating photo-realistic avatars of real people and provide a series of studies to demonstrate their psychological characteristics. We show that avatar faces of familiar people are recognised with high accuracy (Study 1), replicate the familiarity advantage typically observed in real-world face matching (Study 2), and show that these avatars produce a similarity-space that corresponds closely with real photographs of the same faces (Study 3). These studies open the way to conducting psychological experiments on visual perception and social cognition with increased realism in VR.

## Introduction

Over the last decade, Virtual reality (VR) has been increasingly utilised for psychological research (Loomis et al., 1999; McCall & Blascovich, 2009; Wilson & Soranzo, 2015). VR is now commonly deployed by researchers to immerse participants into environments that are increasingly realistic, but which are also highly controlled and consistent for each subject. A key advantage of utilising VR in this manner is that it enables researchers to study human behaviour across a broad range of scenarios that were previously impossible to simulate effectively within the laboratory (Kane et al., 2012). As such, VR enables the study of ‘real-world’ human behaviour from within the confines of the laboratory, by preserving the controlled nature of psychological experiments whilst capturing the realism of more complex environments and social interaction factors.

Despite its growing popularity, one aspect of VR that has so far received limited attention in psychology is the realism of its avatars, and the extent to which they visually represent real people. This is remarkable considering the ubiquity of the human face as a research stimulus in cognitive, developmental, forensic, and social psychology, and in neuroscience and neuropsychology (Bate, 2012; Bruce & Young, 1998; Hole & Bourne, 2010; Bindemann & Megreya, 2017; Rhodes et al., 2011). In cognitive psychology, for example, faces are employed to study processes such as person identification (Bate & Murray, 2017; Bruce & Young, 1986; Fysh & Bindemann, 2017; Johnston & Edmonds, 2009; Ramon & Gobbini, 2018; Young & Burton, 2017), the allocation of visual attention (Langton et al., 2008; Ro et al., 2001), perspective taking (Hermens & Walker, 2012; Langton et al., 2006), and the recognition of emotional states (Keane et al., 2002; Morris et al., 1998; Zhou & Jenkins, 2020).

VR opens up exciting new avenues for knowledge gain in all of these areas, considering that face stimuli are typically presented to participants in laboratory experiments as simplified and disembodied two-dimensional images on a computer screen. This differs from everyday social interaction, where faces are encountered as three-dimensional and highly dynamic stimuli, in diverse and meaningful contexts, and rarely occur in isolation. Some studies have attempted to address this discrepancy between the laboratory and the real world by employing videos of faces (Hermens & Walker, 2012; Keemink et al., 2020; Lander et al., 2001; O’Toole et al., 2011) or by recruiting live confederates to act as stimuli (Kemp et al., 1997; Megreya & Burton, 2008; Ritchie et al., 2020; White et al., 2014), but such approaches come with their own limitations. For instance, while pre-recorded videos display dynamic faces, these representations are seldom interactive. For live confederates, on the other hand, it is challenging to behave consistently across participants, as is necessary to preserve key experimental manipulations. There is also, of course, a limit to the number of live confederates one can employ for a given study.

With VR it is possible to overcome these obstacles by importing digital people—referred to as *avatars*—into virtual environments. These avatars can be programmed to display a wide range of behaviours consistently for each participant. However, the faces of avatars that have been used in some psychological VR studies of face perception bear limited resemblance to real-life faces. For example, some studies have employed avatars that were constructed using synthesised combinations (morphs) of head scans (Bailenson, et al., 2008a; Bülthoff et al., 2019), or from 2D photographs of real people, so that the face shape and texture of the person upon whom they are based are captured poorly (Tummon et al., 2019, 2020). While studies such as these demonstrate that avatars can be useful research stimuli, the faces of these avatars do not resemble real-life faces well.

This is perhaps surprising considering that the feasibility of developing realistic avatars has been demonstrated for many years in the gaming industry, with faithful avatar recreations of real-life people (e.g., Electronic Arts, 2019). Rapid advances in computer science have also shown that 3D representations of faces and bodies can be extracted from 2D photographs via photogrammetry (Bente et al., 2014a, 2014b; Jeni et al., 2017; Narang et al., 2017a; Suwajanakorn et al., 2014), and full body scans can be acquired using structured light and motion sensors (Lucas et al., 2016; Narang et al., 2017b; Shapiro et al., 2014a, 2014b). Whilst some psychology studies have begun to employ these methods to create avatars incorporating higher degrees of realism (Latoschik et al., 2017; Narang et al., 2017a), many behavioural scientists still do not have access to such resources, perhaps because such high-realism avatars and their construction methods have been developed primarily with the skill sets of game designers, 3D artists, and computer scientists in mind.

The construction of avatars with realistic faces for psychological experiments in VR is important practically, as the wider adaptation of this method continues to grow rapidly. The theoretical importance of constructing realistic avatars is also difficult to understate. Research on social interaction, for example, has shown that face-related cognitive processes, such as the attentional engagement and shifting by another person’s eye gaze, vary across controlled laboratory tasks and more realistic paradigms (Cole et al., 2016; Hayward et al., 2017; Skarratt et al., 2012). Thus, avatars that more closely capture real faces will not only improve the quality of the visual experience in VR, but should improve the theoretical relevance of these experiments, by creating a closer correspondence between artificial laboratory settings and real life.

In this paper, we present a method for creating avatars with photo-realistic faces for psychological experiments. These are created by recording 3D scans of the faces of real people with an inexpensive handheld device, and the post-processing and attaching of these scans to animated avatars (i.e., *rigging*) is achieved using widely available graphics software. We provide an overview of the construction process of these avatars, which is accompanied by a comprehensive manual that describes a step-by-step guide for creating such avatars for VR, and which is freely available to download. We employ this approach to construct a set of 120 avatars with photo-realistic faces, and report three studies that demonstrate the potential of these as research stimuli, by showing high recognition rates for avatars with the faces of familiar people (Study 1) as well as a familiarity advantage for the matching of avatars to face photographs (Study 2). We also demonstrate that these avatars produce a similarity-based face-space that closely resembles that of the real people upon whom they are based (Study 3).

## Summary of face scan and avatar construction

We recruited 120 participants (55 male, 65 female) of various ethnicities and a range of ages (mean age = 32 years; *SD* = 13.5; range = 18–86) to have their faces scanned in 3D. Each session proceeded as follows. Using a high-quality digital camera (Fujifilm FinePix S2980, 14-megapixel), we collected a passport-style portrait photograph of each person in a frontal pose, with neutral expression, and under good lighting.

Next, participants were seated and instructed to maintain gaze on a wall-mounted fixation point whilst assuming a relaxed neutral expression. Whilst seated in this position, each subject was scanned using a handheld 3D scanner (Artec Eva). The acquisition of each face scan took approximately two minutes. To process each scan, first, the raw scan was fused into a single wireframe mesh that represented the subject’s head geometry, followed by the application of texture. Following this step, each head scan was ‘wrapped’ to a standardised base geometry, which produced standard UV texture maps and a common 3D topology for each identity.

For body rigging, each person’s head geometry was wrapped onto a standard body mesh. Each avatar was then imported into body-editing software, to be dressed and adapted in terms of height and weight. The avatars’ body shapes and proportions were guided by the structure of their 3D head scan.

**Fig. 1:**
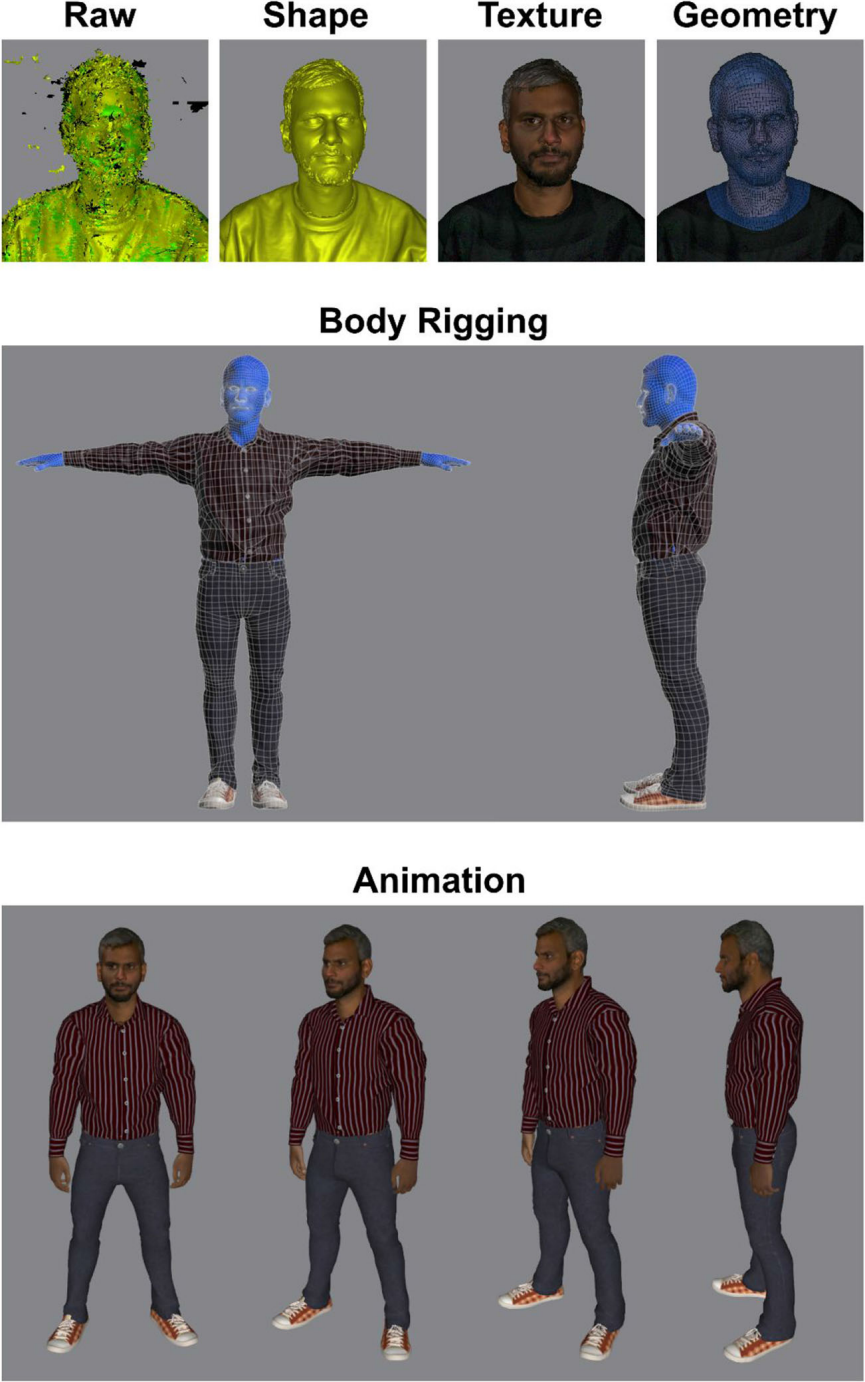
An illustration of the avatar construction process. The upper four images depict the processing of 3D head scans. The middle panel represents the process of attaching the head scan to an avatar body that was created separately. The final panel illustrates the fully animated VR-ready avatar.

**Fig. 2:**
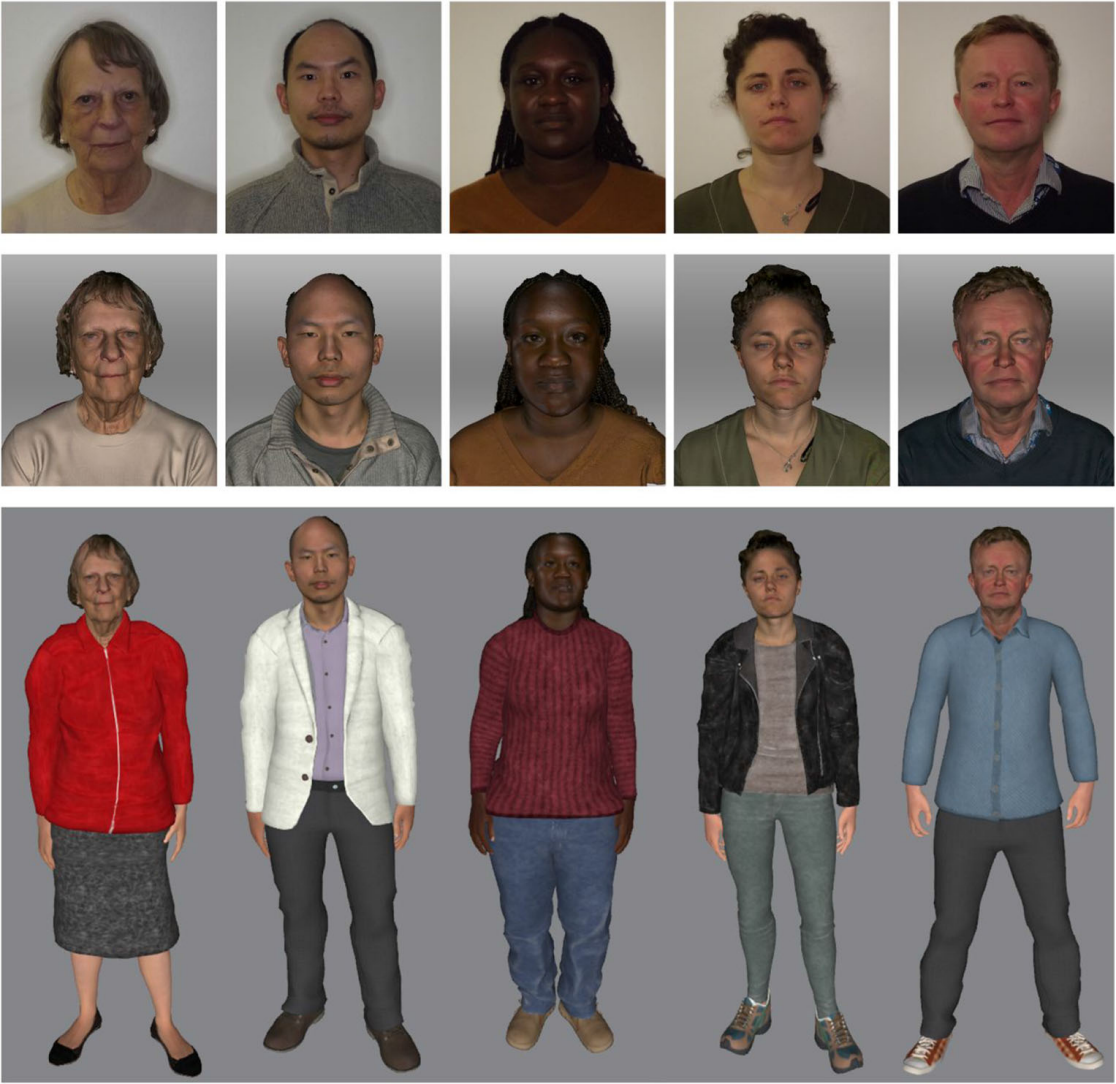
Example identities from the stimulus set, comprising of face photographs (top), 3D scans (middle), and avatars (bottom).

Finally, the avatars were animated using automatic skeleton rigging software to display various idle animations, as well as sitting, standing, turning, and walking, before being integrated into VR. An illustration of this construction workflow is provided in Fig. 1. Examples of avatars, alongside their 3D head scans and digital photographs, are shown in Fig. 2. For researchers wishing to create avatars using our method, we have produced a manual detailing the full construction process, from scanning a person’s head to importing a completed avatar into immersive VR. This manual, along with a time-lapse video of the process, is available for download from https://www.kent.ac.uk/school-of-psychology/vr-avatars/.

## Study 1

We conducted three studies to examine the psychological properties of the avatar faces. These focused on identification of the avatar faces to determine the behavioural correspondence of the face scans with real faces. The first study examined the recognition of avatars, to determine whether these could be identified by people who are familiar with their real-life counterparts. For this purpose, we recruited subjects who would be familiar with a subset of the people that were scanned into our stimulus set. Participants viewed the avatars individually to determine if they could be identified. This was followed by a corresponding identification test with the digital photographs of each person in our stimulus set, and a familiarity check in the form of a name recognition task. If the avatars reliably capture the identity of the people upon whom they are based, then observers who are familiar with these people in real life should also be able to recognise their avatars. If these avatars are to be a useful resource for psychological experiments using familiar identities in VR, then this represents an important first step towards establishing the perceptual properties that these avatars exhibit.

## Method

### Participants

Fifteen participants (10 female, 5 male) with a mean age of 33 years (*SD* = 9.4) were recruited to participate in this study. Because the aim of this study was to investigate familiar face recognition for avatars, we approached individuals who were either staff or former staff at the School of Psychology at the University of Kent, and who would thus be familiar with a subset of our stimuli. None of our participants were featured in the experiment as stimuli. This study was approved by the Ethics Board of the School of Psychology at the University of Kent and was conducted in accordance with the guidelines stipulated by the British Psychological Society and the Helsinki Declaration.

### Stimuli and procedure

This study was conducted during the COVID-19 global pandemic, preventing in-person testing. To overcome this issue, the three tasks that feature in this study were run on a remote computer and screen-shared with participants via telecommunications software (Zoom), which prevented us from obtaining response time data. Participants completed all three tasks (avatar recognition, photo recognition, name recognition) by providing verbal responses (e.g., familiar/unfamiliar), which were then registered by the experimenter as button presses on a standard computer keyboard. The order of Tasks 1 and 2 was counterbalanced across participants, whereas Task 3 was always completed last and served as a familiarity check. These tasks are described below.

### Task 1: Avatar recognition task

**Fig. 3:**
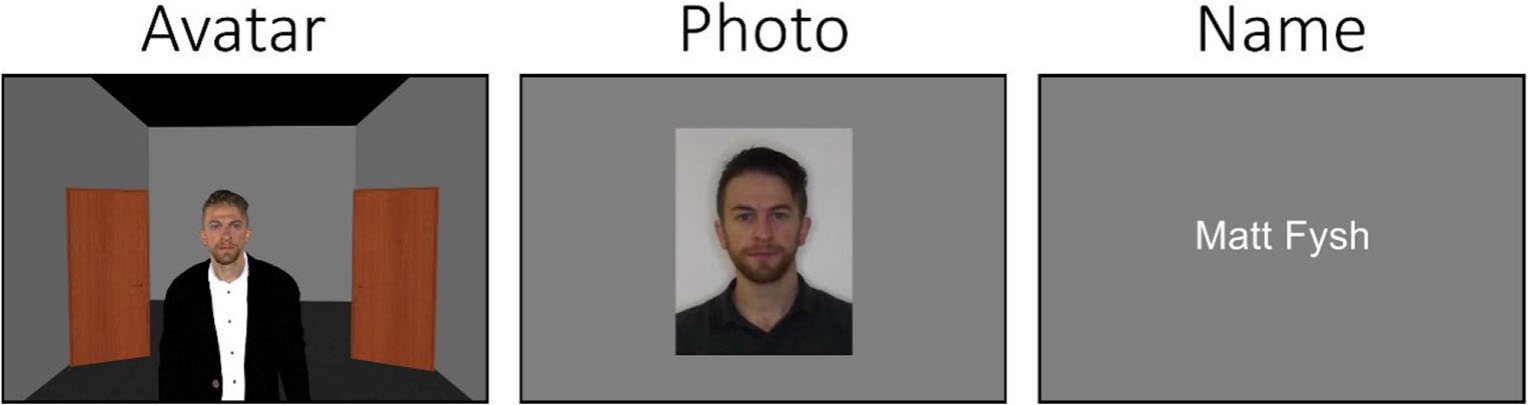
An example trial from each of the three tasks that were employed in Study 1.

The avatar recognition task was presented using Vizard 6 software and featured the 120 avatars that currently make up the avatar stimulus set. The task began by rendering an empty room from a first-person perspective which featured two doors built into the left- and right-hand walls. Each trial began with an avatar entering the room via one door to approach the observer. The avatar would then wait in an ‘idle mode’ until a response was submitted (see Fig. 3). Observers were instructed to verbally confirm recognition of each avatar by way of providing either a name or unique semantic information which would indicate familiarity. Responses were entered manually via one of two button presses by the experimenter on each trial. Upon submission of a response, the avatar exited the room through the other door, thereby triggering the onset of the next trial. The order of avatars was randomised for each observer.

### Task 2: Photograph recognition task

The photograph recognition task was presented using PsychoPy3 software (Peirce, 2007). In terms of procedure, this task was identical to the avatar recognition task, except that the stimuli for this task were digital face photographs of the 120 models in our stimulus set, in which each person was facing forwards with a neutral expression. The photos were cropped and resized to measure 263 (w) × 338 (h) pixels at a resolution of 72 ppi and were presented sequentially in random order.

### Task 3: Name recognition task

The final task was also presented using PsychoPy3 software (Peirce, 2007), and served as a familiarity check for each participant. Observers viewed the name of each person in our avatar database and were instructed to indicate if they were familiar with that person. For some individuals in our stimulus set, nicknames or other aliases were provided alongside given names when relevant. Names were presented one at a time and in a random order for each observer. Given that different observers would be familiar with different subsets of our avatars, the purpose of this task was to enable us to distinguish avatars of familiar from unfamiliar people for each individual observer (for similar approaches, see Bindemann et al., 2017; Burton et al., 1999; Jenkins & Kerr, 2013).

## Results

In the familiarity check, observers reported visual familiarity with an average of 44.5 out of 120 identities (*SD* = 10.0; range = 20–59) in our stimulus set. Of these, 39.1 (*SD* = 9.4) avatars and 40.9 (*SD* = 9.7) photographs were identified on average. Thus, out of the known identities, 87.6% (*SD* = 7.6) were recognised as avatars and 91.9% (*SD* = 6.4) were recognised from photographs.

**Fig. 4:**
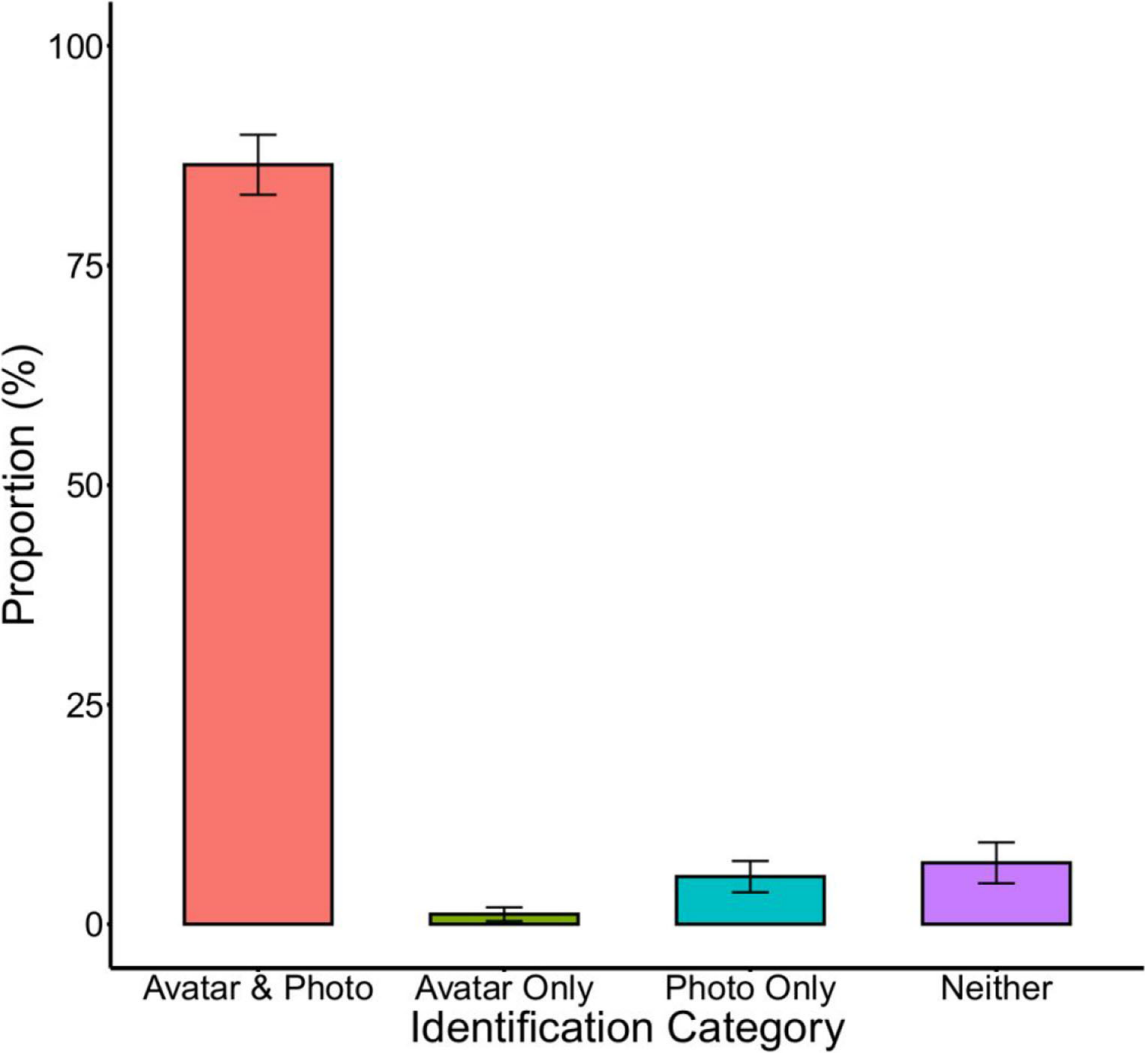
Proportion of response categories in Study 1. Error bars depict the within-subject standard error of the mean (see O’Brien & Cousineau, 2015).

These identification rates were divided into four conditions, reflecting instances for which (i) both the avatars *and* their photograph was recognised, (ii) cases in which the avatar of a person was recognised but *not* their photograph, (iii) cases in which the photograph of a person was recognised but *not* their avatar, and (iv) cases where observers failed to identify someone’s avatar *and* their photograph despite reported familiarity. These data are illustrated in Fig. 4 and were compared via a one-factor repeated-measures analysis of variance (ANOVA), which revealed an effect of condition, *F*(3, 42) = 643.79, *p* < .001, Ƞp^2^ = .98. Tukey’s honestly significant difference (HSD) confirmed that identification rates were substantially higher for both avatar and photograph than for avatars alone, *t*(42) = 37.30, *p* < .001, photos alone, *t*(42) = 35.42, *p* < .001, and cases where neither avatars nor photographs were recognised, *t*(42) = 34.73, *p* < .001. None of the other comparisons were significant, all *t*s ≤ 2.56, all *p*s ≥ .07.

## Discussion

This study compared recognition rates for avatars with 3D face scans and digital photographs of the same people. Identification rates for both sets of stimuli were high, at around 88% for avatars and 92% for photographs. Recognition rates for avatars and photos converged strongly as observers recognised a high proportion of people from their photographs and the corresponding avatars (86%). In turn, cases in which the photograph of a person was recognised, but not their avatar, were low (5%) and did not differ reliably from those cases where an avatar but not its photo was identified (1%). This indicates that the construction method of the avatar faces captures the identity of real people well.

## Study 2

In order to explore the quality of the avatar faces further, we conducted a second study in which observers viewed avatar-photo pairings of familiar and unfamiliar people to decide whether these showed the same person. In psychology, this task is typically referred to as *face matching* and has been studied extensively in recent years with pairs of face photographs (Bindemann, 2021; Burton et al., 2010; Fysh & Bindemann, 2018). In this task, familiarity confers a performance advantage, whereby the faces of known people are matched more accurately than unfamiliar faces (Bruce et al., 2001; Clutterbuck & Johnston, 2005; Jenkins et al., 2011; Megreya & Burton, 2007; Ritchie et al., 2015; Young et al., 1986). Study 2 examines whether this familiarity advantage emerges also when the faces of avatars are matched to photographs.

## Method

### Participants

Twenty participants (4 male, 16 female) with a mean age of 35 years (*SD* = 8.7) were recruited to participate in this study. As in Study 1, we approached members (or former members) of the School of Psychology at the University of Kent, who would thus be familiar with a subset of our avatar stimuli. None of our participants had taken part in Study 1, and none of these featured as stimuli in our stimulus set. This study was approved by the Ethics Board of the School of Psychology at the University of Kent and was conducted in accordance with the guidelines stipulated by the British Psychological Society and the Helsinki Declaration. This study was preregistered at https://osf.io/dcqma.

### Stimuli and procedure

**Fig. 5:**
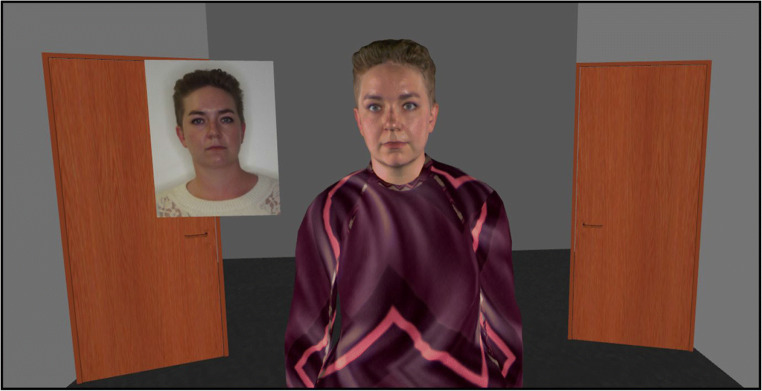
An example match trial from Study 2, depicting an avatar and their corresponding digital face photograph.

As in Study 1, this study was streamed to participants via telecommunications software. The matching task was run using Vizard 6 software. Stimuli for this task comprised of 80 avatar-photo pairings, of which 40 pairings displayed the same person, and the remaining 40 depicted two different people. Upon initiation of the experiment, the participants were presented with the same room as in Study 1. On each trial, an avatar would enter the room through one door and approach the participant. A digital photograph would then appear next to the avatar with approximately similar facial dimensions, which would display either a photograph of the same person or of a different identity (see Fig. 5). Observers classified each pairing verbally as depicting the ‘same person’ or ‘different people’, and these responses were entered by the experimenter via button presses. The avatar then exited the room via the second door, thereby triggering the next avatar to enter. The order of avatar-photo presentation was randomised across observers. Participants then completed a familiarity check which was run in PsychoPy3 (Peirce, 2007), and which entailed viewing the name of each person in our stimulus set and indicating whether or not they were familiar with that person’s visual appearance. As in Study 1, the purpose of this task was to enable us to distinguish familiar from unfamiliar trials for each individual observer given that each person would be familiar with different subsets of avatars. Responses obtained from this task were then used to calculate accuracy for familiar and unfamiliar trials (for similar approaches, see Bindemann et al., 2017; Burton et al., 1999; Jenkins & Kerr, 2013).

## Results

The familiarity check revealed that observers were familiar with, on average, 39.5 out of 120 identities (*SD* = 10.1; range = 22–61) in the stimulus set. These familiarity responses were used retrospectively to classify each avatar-photo pairing as either familiar or unfamiliar for every individual observer. This revealed that of the 80 face pairings that were constructed from our 120 identities, observers were familiar with 34.3 of these on average (*SD* = 7.8; range = 20–50), corresponding to a mean of 42.9% (*SD* = 9.7) of face pairings that were familiar to observers.

Next, each participant’s responses on the naming task were used to divide avatar-photo pairings from the matching task into ‘unfamiliar’ and ‘familiar’ trials, whereby the latter corresponded to identity pairings in which one or both identities (in the case of mismatches) were known to participants. Inspection of these data revealed that of the 80 identity pairings that were viewed by subjects, 19.2% corresponded to familiar match trials on average (*SD* = 4.7; range = 11–28), and 23.7% were familiar mismatch trials (*SD* = 5.6; range = 14–35). Participants’ responses to familiar and unfamiliar face pairings were then converted into the percentage of correct trials, and the cross-subject means were calculated for these conditions. These data are summarised in Table 1.

**Table 1:** Face matching performance in Study 2, with parentheses showing within-subject standard error of the mean (see O’Brien & Cousineau, 2015).

	*Familiar*	*Unfamiliar*	*F - U*
Matches	98.6 (2.16)	96.9 (2.59)	1.72
Mismatches	90.8 (2.95)	84.2 (3.92)	6.67
Sensitivity	3.14 (0.15)	2.81 (0.15)	0.33
Criterion	−0.18 (0.04)	−0.34 (0.04)	0.16

A 2(familiarity: familiar vs. unfamiliar) × 2(trial: match vs. mismatch) within-subject ANOVA of these data confirmed a main effect of familiarity, *F*(1, 19) = 5.99, *p* < .05, Ƞ_p_^2^ = .24, due to higher accuracy for familiar face trials (*M* = 94.7%, *SD* = 9.3) than unfamiliar trials (*M* = 90.5%, *SD* = 12.6). An effect of trial type was also found, *F*(1, 19) = 13.39, *p* < .01, Ƞ_p_^2^ = .41, due to higher accuracy on match trials (*M* = 97.7%, *SD* = 3.6) versus mismatch trials (*M* = 87.5%, *SD* = 13.6). The interaction of these factors was not significant, *F*(1, 19) = 2.44, *p* = .14, Ƞ_p_^2^ = .11.

These accuracy data were also converted to sensitivity and *criterion* (see Table 1), using the log-linear calculation method (Hautus, 1995; Stanislaw & Todorov, 1999). Together, these measures provide a bias-free index of overall performance (i.e., sensitivity), as well as participants’ response patterns (i.e. *criterion*), respectively (Stanislaw & Todorov, 1999). A paired samples *t*-test showed that sensitivity was higher on familiar trials compared to unfamiliar trials, *t*(19) = 2.18, *p* < .05, *d* = .49. *Criterion* also varied between the two levels of familiarity, *t*(19) = 2.57, *p* < .05, *d* = .57, indicating a greater tendency to classify unfamiliar pairings as identity matches, compared to familiar face pairings.

## Discussion

This study provides converging evidence that the avatars capture the identity of the people upon whom they are based. This was characterised by near-ceiling accuracy on trials with familiar faces (95%) as well as greater sensitivity for familiar face pairings than unfamiliar pairings. These results align with those of Study 1, by showing that our avatars can be recognised as their real-life counterparts. In this study, this advantage is characterised by a matching advantage for familiar over unfamiliar face pairings, which has also been demonstrated previously for pairs of face photographs (Clutterbuck & Johnston, 2005; Jenkins et al., 2011; Lander et al., 2001; Megreya & Burton, 2007; Ritchie et al., 2015; Young et al., 1986).

An advantage for match trials was also observed, whereby observers were generally better at detecting that an avatar and photograph depicted the same identity (98%) than when an avatar and photograph depicted different identities (88%). This was corroborated by a response bias to classify both face pairings as identity matches, with a greater tendency towards this response option when viewers were unfamiliar with the identity depicted. This would be consistent with other studies in which observers matched face photographs to live people and exhibited similar response patterns that were indicative of biases towards ‘same identity’ classifications (Kemp et al., 1997; Megreya & Burton, 2008; Ritchie et al., 2020). On the other hand, it may also simply be that the match trials were easier to classify than the mismatch trials. This explanation is plausible considering that the face scan upon which each avatar was based was obtained only a few minutes after the digital face photograph was acquired – a method which is known to boost the correspondence of photographs in face-matching experiments substantially (Megreya et al., 2013).

## Study 3

In this final study, we examined the similarity space of the set of faces, to establish whether the people who look similar (or different) in their photos, also look similar (or different) in their avatars. By quantifying the similarity between individuals, it is possible to examine whether the overall set of relations between faces is preserved as we move from photos into VR.

Principal components analysis (PCA) is a popular technique for representing the ‘space’ spanned by a set of faces and is used both for automatic recognition purposes and for understanding human face perception (Burton et al., 2016; Kirby & Sirovich, 1990; Phillips et al., 2000; Turk & Pentland, 1991). In typical use, PCA takes a large number of face images and derives a relatively small number of dimensions, within which any face can be described, either as a set of coordinates or (equivalently) a weighted sum of eigenvectors. An introduction to the technique can be found in Valentin et al. (1994), and a freely available software package supporting PCA on face sets, InterFace, is described in Kramer et al. (2017).

Here, we applied PCA to two sets of face images, one based on photos of our volunteers, and one based on their avatars. Within such spaces, faces can be described as more or less similar to each other according to how close they lie, whereby similar faces will be nearer within the PCA space and dissimilar faces will be farther apart. We compared ‘photo-space’ and ‘avatar-space’ by comparing all the pairwise distances between individuals in the two spaces.

## Method

**Fig. 6:**
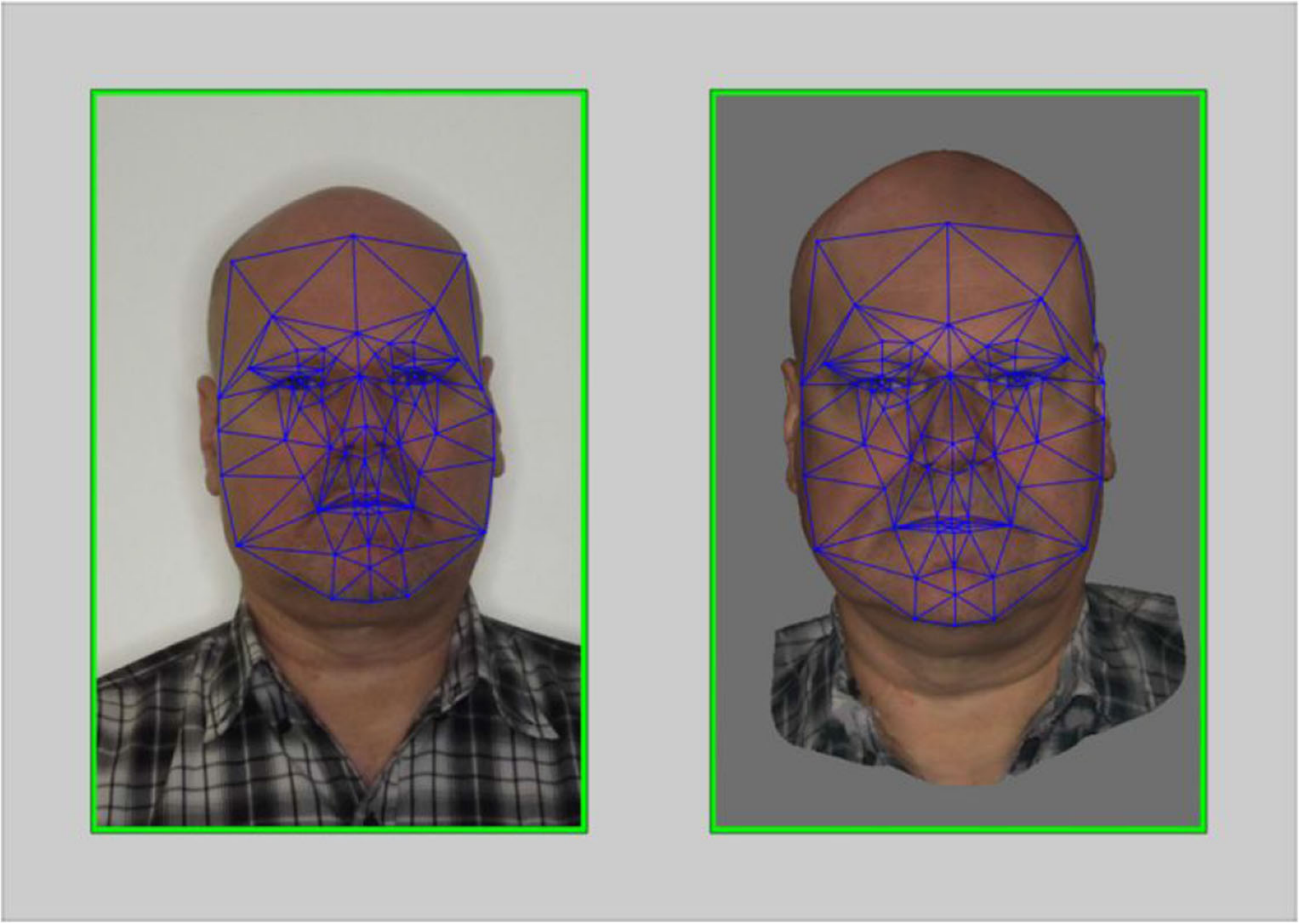
An illustration of the 82 fiducial points that were assigned to digital face photographs and 3D scans of faces during the landmarking process.

Separate PCA analyses were conducted on the 120 photos and 120 avatar images described in Studies 1 and 2, using the InterFace software package (Kramer et al., 2017). Prior to analysis, all images were shape-standardised by morphing them to the InterFace template, based on 82 fiducial points for each image (e.g., corners of the eyes, corners of the mouth, etc.). Assignment of the fiducials was carried out using a standard semi-automatic process requiring five manually aligned landmarks (see Kramer et al., 2017, for details). An illustration of this landmarking process is visualised in Fig. 6. PCA was then computed on these normalised images. Within each PCA-derived space (photos and avatars), Euclidean pair-wise distances were calculated for all combinations of faces.

## Results

**Fig. 7:**
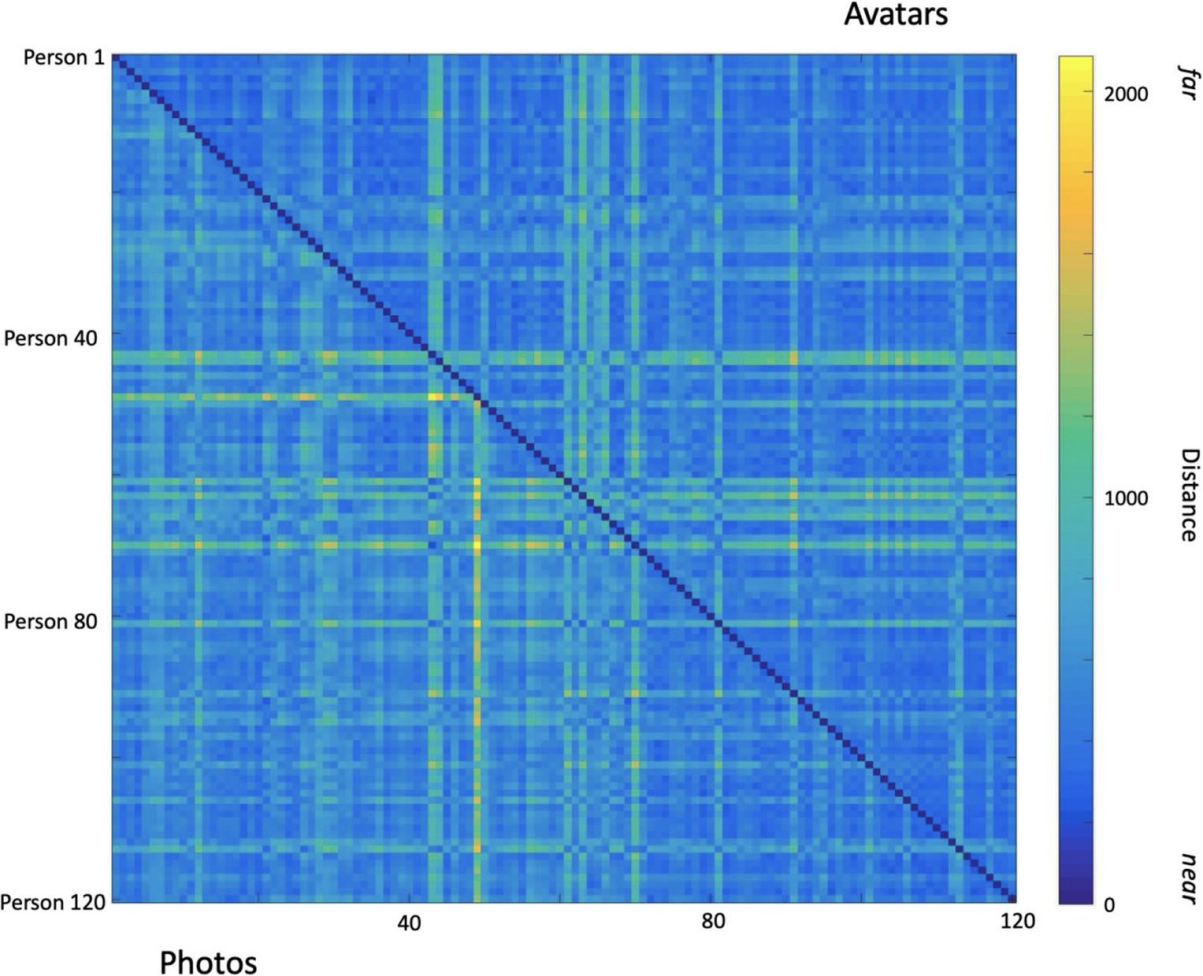
Similarity matrices for photos (lower left triangle) and avatars (upper right triangle). Persons 1 through 120 are depicted along the *x*-axis, from left to right. For the *y*-axis, Persons 1 through 120 are depicted from top to bottom. Units denote Euclidean distance in 119-dimensional PCA-space.

Figure 7 shows similarity matrices for pairs of face photos and pairs of avatars. It is clear from this image that there are some faces which are relatively similar to many others, represented by predominantly blue columns/rows. There are also some faces which are generally dissimilar to most other faces, represented by predominantly green and yellow columns/rows. To compare the two spaces, imagine a fold along the identity-diagonal (where faces have zero distance to themselves). The two similarity matrices appear highly symmetrical, whereby a person whose photo seems unlike most others also has an avatar with the same property. Visually, these similarity matrices therefore imply a very high degree of correspondence.

To test the similarity of the two distance matrices, we computed a correlation between the values in the matrices. This showed a very high degree of association; Pearson’s *r*(7078) = .64, *p* < .001. To guard against any potential skewing effects, we also report the non-parametric correlation coefficient, Spearman’s *rho*(7078) = .59, *p* < .001.^1^

The similarity matrices in Fig. 7 represent distances within 119-dimensional space (i.e., the maximal span produced by PCA). However, as noted previously, PCA is usually used to compress stimuli into a smaller number of dimensions than the original set. This is possible because the technique extracts dimensions in order, such that early components capture most variance in the set. In order to test similarity space within a more compressed dimensional range, we repeated the process above, using only the first 30 dimensions derived from the PCA on photos and avatars. This produced almost identical results, with correlations between the two similarity matrices being almost unchanged (Pearson’s *r* = .63; Spearman’s *rho* = .59).

## Discussion

This study demonstrates a high degree of similarity between the ‘face-space’ derived from photos of our 120 volunteers and the space derived from their avatars. Once again, this represents strong support for the claim that the avatars we have created preserve the identity information that is provided in the corresponding photographs of their real-life counterparts.

We should note that this analysis adds a very useful source of evidence over Studies 1 and 2. In those studies, we rely on viewers who are familiar with some of the people whose images they are shown. It is well established that familiar viewers are excellent at face recognition, even when stimuli comprise severely degraded, poor-quality images (Bruce et al., 1999, 2001; Jenkins & Kerr, 2013). So the fact that humans can recognise the avatars of people they know provides only some evidence for a good correspondence between photographic representations and representations in VR—any loss of information caused by a move to the virtual world may be compensated by our excellent ability to recognise familiar people. However, in Study 3, there is no reliance on familiarity. Instead, this comparison is based entirely on statistical analysis of physical, pixel-by-pixel properties of the photos on the one hand and the avatars on the other. The high degree of correspondence shown in Fig. 7 demonstrates that, at a detailed level, similarity structure between a set of 120 people is highly consistent regardless of whether they are represented by a particular photo or by an avatar. This adds a further strand to the evidence supporting the utility of avatars for studying face perception in VR.

## General discussion

Across three studies, we demonstrate the perceptual properties of 120 avatars that were rigged with head scans of real people. Study 1 demonstrates that prior familiarity with an individual facilitates recognition of an avatar that is constructed from a 3D scan of that person’s head. These recognition rates largely coincided with photographic identification of the same people, implying that our avatars preserve sufficient identity-relevant information to facilitate recognition. In addition, the results of Study 2 show that familiarity confers an advantage in terms of both accuracy and sensitivity for matching avatar-photo pairings when compared against pairings with whom subjects were unfamiliar. These results converge with previous work comparing performance for matching familiar versus unfamiliar photo-photo pairings, which give rise to very similar patterns of accuracy (Bruce et al., 2001; Clutterbuck & Johnston, 2005; Jenkins et al., 2011; Megreya & Burton, 2007; Noyes & Jenkins, 2019; Ritchie et al., 2015; Young et al., 1986). Finally, the PCA employed in Study 3 revealed a high degree of similarity between the perceptual space occupied by our avatar stimuli and their photographic counterparts, further demonstrating that the avatars adequately preserve the identity information that is conveyed in photographs. Together, these studies reflect that our avatars are processed similarly to photographs of faces. This important convergence of results reflects that, like photographs, our avatars can be utilised as stimuli for exploring questions pertaining to person perception in VR. However, unlike photographs, the dynamic nature of our avatars means that these should present useful stimuli for investigating avenues of research that were previously unavailable to examine based on static images alone.

There are several reasons for anticipating that the construction of photo-realistic avatars represents a timely development for researchers seeking to investigate human behaviour in VR. First, studies have demonstrated that the realism of avatars is important for understanding human behaviour. Experiments on visual perspective taking, for example, show that humanness and the correspondence between avatar and participant affect interpretation of what the avatar can see (Ferguson et al., 2018; Nielsen et al., 2015). Second, by following our avatar construction method, it is possible to import avatars of real people into VR. This is important for studying questions pertaining to the perception of facial identity, which represents a mainstream field of study spanning multiple psychological domains (see, e.g., Bate, 2012; Bindemann, 2021; Rhodes et al., 2011). The limited realism of such avatars that have been employed in VR studies of face perception thus far (Bülthoff et al., 2019; Tummon et al., 2019, 2020) makes it difficult to draw firm conclusions about how faces are processed in the real world. In line with this reasoning, there is evidence in support of the notion that the more closely avatars resemble real humans, the more likely these are to elicit neural and behavioural responses similar to those evoked by actual people (see de Borst & de Gelder, 2015). Finally, our construction method also holds great potential for enhancing the theoretical value and real-world applicability of studies interested in examining social interaction in VR, but which were hitherto constrained to using generic avatars to represent real people (Bailenson et al., 2008b, 2008c; Kane et al., 2012; Roth et al., 2015). By creating realistic avatars that are based on actual people and which bear a close correspondence to their real-life counterparts, progress is made towards resolving this barrier between the virtual and physical world.

There remain many aspects in which the realism of our avatars could be enhanced further. Our avatars are rigged with generic movement animations that are not personalised, so that individualistic motion profiles are not captured. Such information has been claimed to carry additional identity cues (Bläsing & Sauzet, 2018; Cutting & Kozlowski, 1977; Loula et al., 2005) that could enhance the realism of avatars in VR (Narang et al., 2017a, 2017b). The faces of our avatars are also static and thus cannot currently convey expressions or articulate speech. Facial rigging is challenging (Grewe et al., 2021; Lewis et al., 2014) and, if poorly implemented, can reduce the perception of behavioural realism (Grewe et al., 2021). Advances in technology and expertise will close this gap, to create avatars that not only visually resemble their real-life counterparts, but which incorporate their speech and motion patterns as well.

Although the successful implementation of these personal aspects will undoubtedly improve avatars, our avatar construction method provides a good starting point for veridically representing real people in VR. This method holds much potential for exploring psychological questions of face and person perception, and we have demonstrated their correspondence to photographic faces in some key perceptual tests. As is the case with photographs, for which there are questions that can *only* be explored using image-bound face stimuli (Bobak et al., 2019; Mileva et al., 2020; Noyes & Jenkins, 2017; Pachai et al., 2017; Sandford & Burton, 2014), there are also many research questions that cannot be pursued via photographs, but which could be investigated using avatars. Such questions would incorporate the dynamic and interactive nature of avatars in VR, thereby allowing one to study aspects of social interaction and person perception under conditions that simulate reality, whilst retaining the control afforded by laboratory settings.

More generally, our avatars also hold broader application beyond that of face perception. For example, our avatar construction method could be used to generate useful stimuli for social psychological questions that can be explored in VR using avatars (see, e.g., Kane et al., 2012; Skulmowski et al., 2014; Slater & Steed, 1999), as well as cognitive studies in which the participant must simulate the visual perspective of somebody else (see, e.g., Begeer et al., 2010; Ferguson et al., 2018). Likewise, clinical research studies are increasingly utilising VR to enhance the real-world application of assessment tools (Bell et al., 2020), within which avatars already represent a key component (Mölbert et al., 2018; Powers et al., 2013). In light of the increasing application of VR as a research tool across these various psychological disciplines (for reviews, see Gaggioli, 2001; Peeters, 2019; Smith, 2019; Wilson & Soranzo, 2015), we hope that our avatars can be useful tools for exploring questions in such research domains.

A key advantage for these domains is that these avatars can be imported into a range of virtual environments. Given the growing body of evidence that the context within which persons are encountered influences how these are perceived (Feng & Burton, 2019; McCaffery & Burton, 2016; Robertson & Burton, 2020), this can be explored further in VR by immersing participants in contexts that simulate relevant applied settings. Some studies have already utilised this approach, for example, by using VR to immerse participants within an airport to match a queue of travellers against their passport photographs (Tummon et al., 2019, 2020). In light of an increasing emphasis on translating laboratory-based findings to real-world contexts in which people represent a key stimulus (De Lillo et al., 2021; Hayward et al., 2017; Ramon et al., 2019), this line of enquiry is only likely to gain further traction as more researchers begin to utilise VR for studying human behaviour. We anticipate that our construction method for VR-ready avatars will prove to be a valuable resource for such work.

## Data Availability

The datasets generated during and/or analysed during the current study are available in the OSF repository https://osf.io/qtv9k/.

## Footnotes

1 On the advice of a reviewer this analysis was also repeated excluding one outlying identity (Person 49). This revealed similar correlation effect sizes of .71 (Pearson) and .61 (Spearman).
